# Supplementary material for: Spatial predictive risk mapping of lymphatic filariasis residual hotspots in American Samoa using demographic and environmental factors
Source: PLoS Negl Trop Dis. 2023 Jul 24;17(7):e0010840. doi: 10.1371/journal.pntd.0010840 (PMC10399813; doi:10.1371/journal.pntd.0010840)
Supplement: S1 Table — Data extracted from the National Weather Services Websiteand average month rainfall from the spatial layer in 2006. (DOCX) [file pntd.0010840.s001.docx]

|  | **Jan** | **Feb** | **Mar** | **Apr** | **May** | **Jun** | **Jul** | **Aug** | **Sep** | **Oct** | **Nov** | **Dec** | **Year total** | **Mean** | **SD** |
| --- | --- | --- | --- | --- | --- | --- | --- | --- | --- | --- | --- | --- | --- | --- | --- |
| **2000** | 341.12 | 343.92 | 463.30 | 123.19 | 340.36 | 201.42 | 84.84 | 81.53 | 171.96 | 281.43 | 229.62 | 264.41 | 2927.10 | 243.92 | 117.61 |
| **2001** | 162.31 | 347.22 | 386.33 | 168.15 | 121.67 | 117.09 | 119.13 | 76.96 | 119.89 | 223.52 | 216.92 | 400.56 | 2459.74 | 204.98 | 113.10 |
| **2002** | 188.47 | 123.19 | 319.02 | 453.14 | 284.48 | 150.37 | 264.67 | 149.86 | 41.15 | 301.50 | 387.60 | 201.68 | 2865.12 | 238.76 | 118.04 |
| **2003** | 188.21 | 231.39 | 264.41 | 236.98 | 497.59 | 195.33 | 315.72 | 172.21 | 109.73 | 164.59 | 543.31 | 425.96 | 3345.43 | 278.79 | 139.22 |
| **2004** | 312.67 | 346.71 | 345.44 | 187.96 | 129.79 | 95.50 | 180.59 | 67.82 | 125.73 | 292.61 | 101.60 | 245.87 | 2432.30 | 202.69 | 102.25 |
| **2005** | 336.55 | 267.97 | 328.68 | 368.30 | 273.81 | 338.33 | 137.92 | 344.42 | 366.52 | 171.45 | 246.13 | 632.21 | 3812.29 | 317.69 | 123.84 |
| **2006** | 606.30 | 743.46 | 215.90 | 130.81 | 199.64 | 128.78 | 210.06 | 222.25 | 402.08 | 247.65 | 408.69 | 549.40 | 4065.02 | 338.75 | 202.00 |
| **2007** | 533.65 | 262.89 | 515.87 | 118.36 | 440.44 | 87.38 | 144.02 | 120.40 | 432.31 | 410.97 | 371.09 | 250.44 | 3687.83 | 307.32 | 163.42 |
| **2008** | 562.61 | 124.46 | 256.03 | 327.41 | 503.68 | 304.80 | 113.28 | 91.19 | 155.70 | 82.04 | 301.50 | 470.15 | 3292.86 | 274.40 | 168.38 |
| **2009** | 288.04 | 193.55 | 253.75 | 235.71 | 486.92 | 92.71 | 252.98 | 264.41 | 159.51 | 191.26 | 422.91 | 695.96 | 3537.71 | 294.81 | 165.77 |
| **2010** | 719.07 | 402.08 | 126.75 | 262.38 | 167.89 | 128.27 | 210.82 | 109.73 | 245.87 | 389.38 | 232.41 | 313.44 | 3308.10 | 275.67 | 169.80 |
| **2011** | 653.29 | 340.11 | 146.56 | 101.85 | 93.73 | 178.05 | 58.67 | 97.79 | 48.77 | 321.82 | 404.11 | 246.13 | 2690.88 | 224.24 | 179.45 |
| **2012** | 206.76 | 298.70 | 355.60 | 213.61 | 308.61 | 155.70 | 148.34 | 81.03 | 323.60 | 126.75 | 466.09 | 465.07 | 3149.85 | 262.49 | 127.79 |
| **2013** | 464.06 | 266.19 | 253.24 | 343.41 | 161.29 | 298.70 | 215.39 | 460.76 | 206.76 | 145.03 | 150.11 | 322.83 | 3287.78 | 273.98 | 109.52 |
| **2014** | 496.32 | 295.91 | 176.53 | 362.97 | 199.14 | 208.79 | 469.39 | 81.79 | 66.04 | 68.07 | 349.50 | 423.67 | 3198.11 | 266.51 | 155.32 |
| **2015** | 682.50 | 310.39 | 174.24 | 364.49 | 421.39 | 230.38 | 101.35 | 171.45 | 30.48 | 210.82 | 542.04 | 597.66 | 3837.18 | 319.76 | 205.61 |
| **2016** | 112.78 | 164.85 | 273.30 | 784.10 | 251.21 | 98.55 | 113.79 | 114.05 | 138.43 | 130.05 | 326.64 | 361.70 | 2869.44 | 239.12 | 194.38 |
| **2017** | 243.08 | 466.34 | 139.70 | 206.76 | 590.04 | 127.25 | 118.36 | 197.61 | 159.00 | 510.79 | 327.41 | 306.32 | 3392.68 | 282.72 | 160.76 |
| **2018** | 390.40 | 824.74 | 161.54 | 451.10 | 235.20 | 82.30 | 245.87 | 247.65 | 218.44 | 304.55 | 302.26 | 573.79 | 4037.84 | 336.49 | 201.91 |
| **2019** | 281.94 | 610.62 | 291.34 | 258.32 | 378.71 | 158.50 | 445.52 | 218.44 | 134.11 | 246.63 | 189.99 | 455.42 | 3669.54 | 305.79 | 141.06 |
| **2020** | 558.04 | 831.34 | 208.03 | 395.22 | 177.04 | 526.80 | 283.72 | 247.14 | 378.46 | 473.20 | 412.75 | 369.57 | 4861.31 | 405.11 | 180.20 |
| Mean | 396.49 | 371.35 | 269.24 | 290.32 | 298.20 | 185.93 | 201.68 | 172.21 | 192.02 | 252.22 | 330.20 | 408.18 | 3368.04 | 280.67 |  |
| **SD** | 187.48 | 210.99 | 105.03 | 156.94 | 145.73 | 107.66 | 110.43 | 101.31 | 122.46 | 122.01 | 119.73 | 140.94 | 581.04 | 48.42 |  |
| **Spatial layer**  **Rainfall** | 374.24 | 366.61 | 347.45 | 325.46 | 323.63 | 264.32 | 221.15 | 220.06 | 233.83 | 341.73 | 338.09 | 377.16 | 3733.73 | 311.14 |  |

**S1 table.** Average monthly rainfall (mm) in the Pago Pago area, in American Samoa from 2000-2020. Data extracted from the National Weather Service Website (1) and average month rainfall from the spatial layer in 2006

**References**

1. National Oceanic and Atmospheric Administration. National Weather Service 2022 [Available from: <https://www.weather.gov/wrh/Climate?wfo=ppg>.
