# Supplementary material for: Spatial predictive risk mapping of lymphatic filariasis residual hotspots in American Samoa using demographic and environmental factors
Source: PLoS Negl Trop Dis. 2023 Jul 24;17(7):e0010840. doi: 10.1371/journal.pntd.0010840 (PMC10399813; doi:10.1371/journal.pntd.0010840)
Supplement: S2 Table — (DOCX) [file pntd.0010840.s002.docx]

**S2 Table.** Odds ratios (ORs) and 95% CrI from the non-spatial models for Ag and Wb123, Bm14 and Bm33 antibodies in a community survey in American Samoa in 2016

| **Model** | **Participants**  **N (%)** | **Antigen** | **Wb123 antibody** | **Bm14 antibody** | **Bm33 antibody** |
| --- | --- | --- | --- | --- | --- |
|  |  | **ORs, posterior mean**  **(95% CrI)** | **ORs, posterior mean**  **(95% CrI)** | **ORs, posterior mean**  **(95% CrI)** | **ORs, posterior mean**  **(95% CrI)** |
| **Total samples** | 2671 | 2671 | 2671 | 2671 | 2671 |
| **Total positives, N (%)** | - | 135 (5.05) | 684 (25.60) | 350 (13.10) | 1219 (45.63) |
| **Gender** |  |  |  |  |  |
| Male  Female | 1209 (45.26)  1462 (54.74) | Ref  0.58  (0.49 to 0.68) | Ref  0.49  (0.41 to 0.59) | Ref  0.46  (0.36 to 0.59) | Ref  0.72  (0.61 to 0.85) |
| **Age (per year)** | - | 0.97  (0.96 to 0.98) | 1.02  (1.02 to 1.03) | 1.03  (1.02 to 1.04) | 1.02  (1.02 to 1.03) |
| **Work location** |  |  |  |  |  |
| Indoor | 727 (27.22) | Ref | Ref | Ref | Ref |
| Outdoor | 40 (1.50) | 1.01  (0.83 to 1.22) | 2.78  (1.41 to 5.47) | 3.24  (1.56 to 6.68) | 2.87  (1.39 to 6.10) |
| Tuna cannery | 131 (4.90) | 0.99  (0.82 to 0.19) | 2.21  (1.46 to 3.32) | 1.94  (1.18 to 3.15) | 1.69  (1.14 to 2.51) |
| Others | 1773 (66.40) | 0.51  (0.44 to 0.60) | 1.50  (1.18 to 1.91) | 1.27  (0.94 to 1.73) | 1.10  (0.89 to 1.35) |
| **Population density (people/m^2^)** | - | 0.89  (0.81 to 0.98) | 0.95  (0.88 to 1.02) | 0.88  (0.79 to 0.97) | 0.95  (0.89 to 1.02) |
| **Elevation (m)** | - | 1.00  (0.99 to 1.00) | 1.00  (0.99 to 1.00) | 1.00  (0.99 to 1.00) | 1.00  (0.99 to 1.00) |
| **Distance to streams (m)** | - | 1.00  (1.00 to 1.00) | 0.99  (0.99 to 0.99) | 0.99  (0.99 to 1.00) | 0.99  (0.99 to 1.00) |
| **Rainfall in the wettest month - December (mm)** | - | 1.00  (0.99 to 1.00) | 1.00  (0.99 to 1.00) | 1.00  (0.99 to 1.00) | 1.00  (0.99 to 1.00) |
| **Land Cover** |  |  |  |  |  |
| Cropland (%) | - | 1.00  (0.94 to 1.05) | 1.04  (0.99 to 1.11) | 0.96  (0.82 to 1.05) | 1.03  (0.98 to 1.11) |
| Trees (%) | - | 1.01  (1.001 to 1.01) | 1.01  (1.001 to 1.01) | 1.01  (1.001 to 1.01) | 1.01  (1.001 to 1.01) |
| Built/Urban (%) | - | 0.99  (0.99 to 1.00) | 0.99  (0.99 to 1.00) | 0.99  (0.99 to 1.00) | 0.99  (0.99 to 1.00) |
| DIC |  | 1122.32 | 2861.15 | 1898.63 | 3505.57 |

*Footnotes: ORs, Odds ratios; 95% CrI, 95% credible interval; DIC, deviance information criterion. Statistically significant ORs are highlighted in blue (positive associations) and grey (negative associations).*
