# Supplementary material for: Spatial predictive risk mapping of lymphatic filariasis residual hotspots in American Samoa using demographic and environmental factors
Source: PLoS Negl Trop Dis. 2023 Jul 24;17(7):e0010840. doi: 10.1371/journal.pntd.0010840 (PMC10399813; doi:10.1371/journal.pntd.0010840)
Supplement: S1 Fig — The driest (August) and wettest (December) months in 2016 were representative of the average rainfall in the respective months in previous 20 years. (DOCX) [file pntd.0010840.s003.docx]

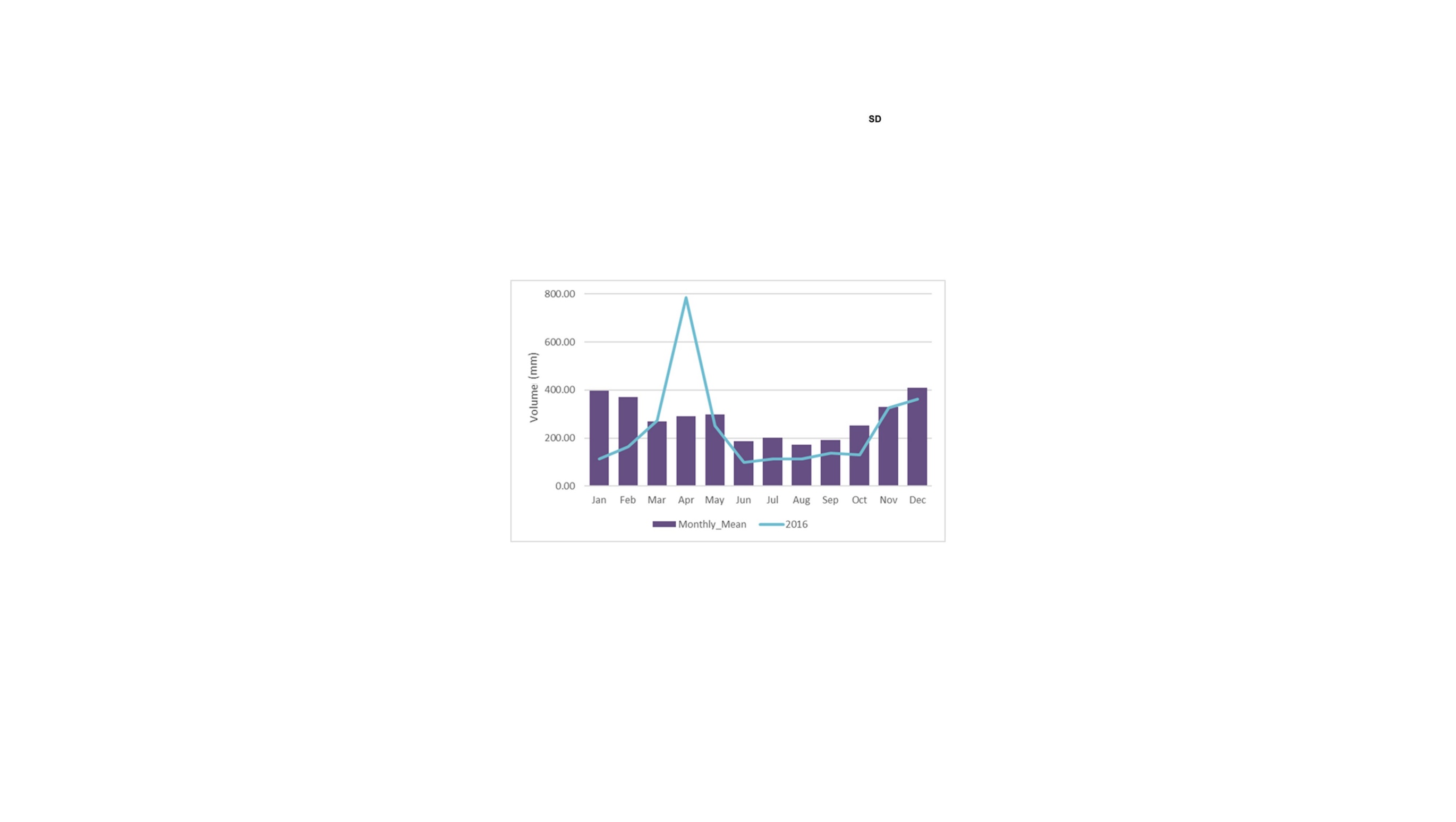


**S1 Fig.** Average monthly rainfall (mm) for the period 2000-2020 and average montly rainfall (mm) in 2016 in American Samoa. The driest (August) and wettest (December) months in 2016 were representative of the average rainfall in the respective months in previous 20 years
