# Supplementary material for: Spatial predictive risk mapping of lymphatic filariasis residual hotspots in American Samoa using demographic and environmental factors
Source: PLoS Negl Trop Dis. 2023 Jul 24;17(7):e0010840. doi: 10.1371/journal.pntd.0010840 (PMC10399813; doi:10.1371/journal.pntd.0010840)
Supplement: S2 Fig — The horizontal line indicates the average in the previous 10 years. Total rainfall in 2016 was representative of average rainfall in the previous 20 years. (DOCX) [file pntd.0010840.s004.docx]

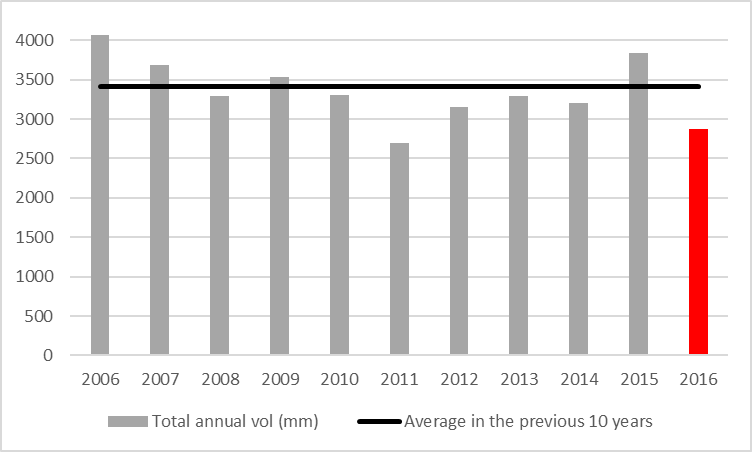


**S2 Fig**. Average annual rainfall (mm) for the period 2000-2020. The horizontal line indicates the average in the previous 10 years. Total rainfall in 2016 was representative of average rainfall in the previous 20 years.
