# Supplementary figures and images for: Spatial predictive risk mapping of lymphatic filariasis residual hotspots in American Samoa using demographic and environmental factors
Source: PLoS Negl Trop Dis. 2023 Jul 24;17(7):e0010840. doi: 10.1371/journal.pntd.0010840 (PMC10399813; doi:10.1371/journal.pntd.0010840)

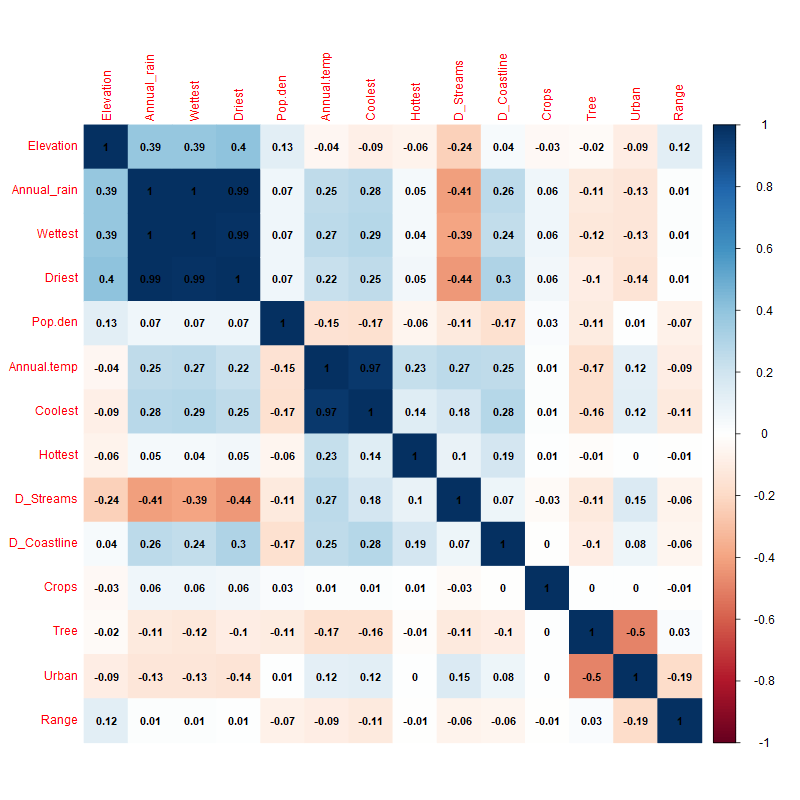


**S3 Fig.** Correlation matrix of the variables extracted for the analysis

Supplement: S3 Fig — (DOCX) [file pntd.0010840.s005.docx]
