# Supplementary material for: Spatial predictive risk mapping of lymphatic filariasis residual hotspots in American Samoa using demographic and environmental factors
Source: PLoS Negl Trop Dis. 2023 Jul 24;17(7):e0010840. doi: 10.1371/journal.pntd.0010840 (PMC10399813; doi:10.1371/journal.pntd.0010840)
Supplement: S4 Fig — (DOCX) [file pntd.0010840.s006.docx]

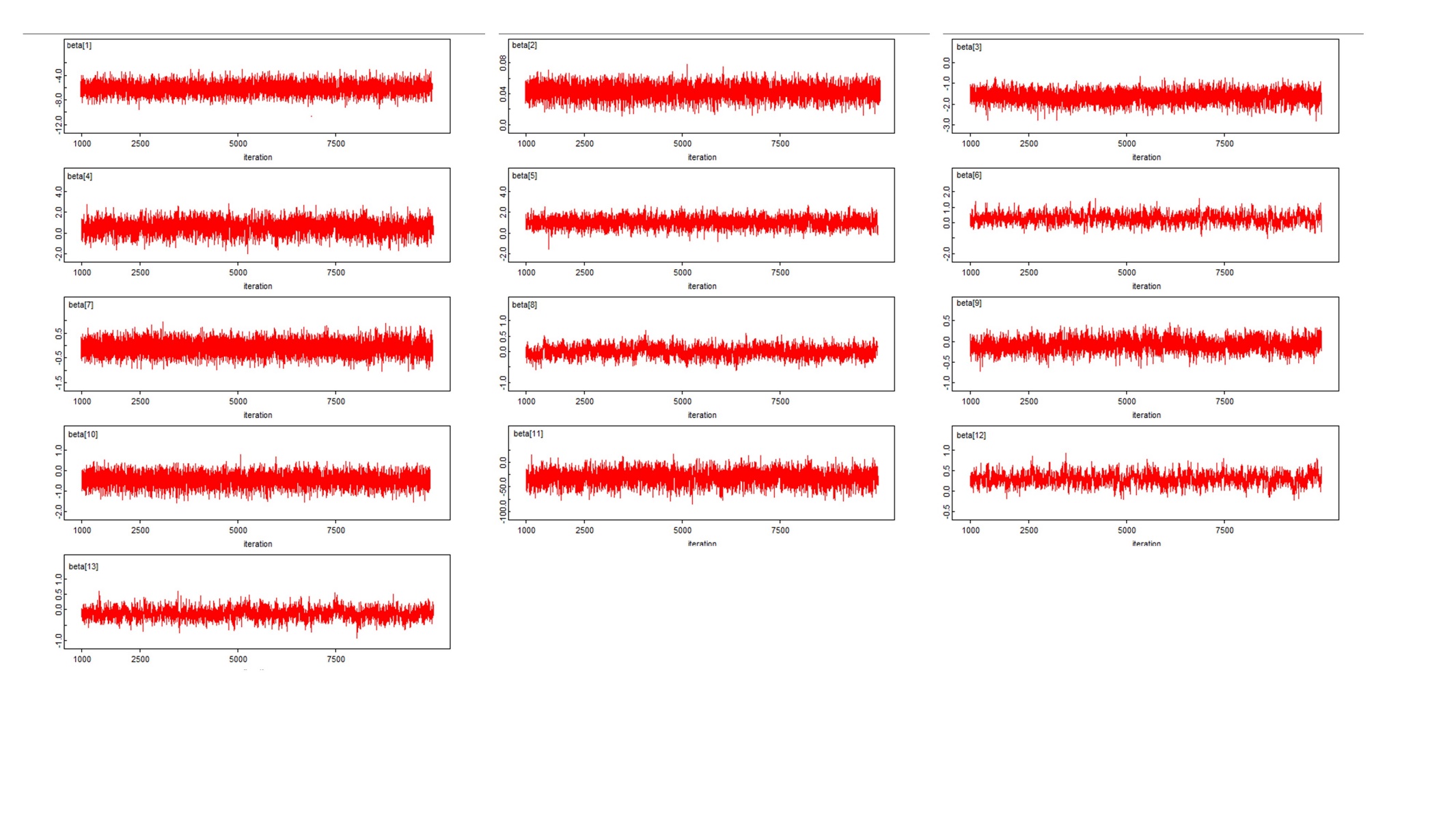


**S4 Fig.** History plots of the last 10,000 values from the posterior distributions of the covariate parameters in the model for Ag
