# Supplementary material for: Spatial predictive risk mapping of lymphatic filariasis residual hotspots in American Samoa using demographic and environmental factors
Source: PLoS Negl Trop Dis. 2023 Jul 24;17(7):e0010840. doi: 10.1371/journal.pntd.0010840 (PMC10399813; doi:10.1371/journal.pntd.0010840)
Supplement: S8 Fig — (DOCX) [file pntd.0010840.s010.docx]

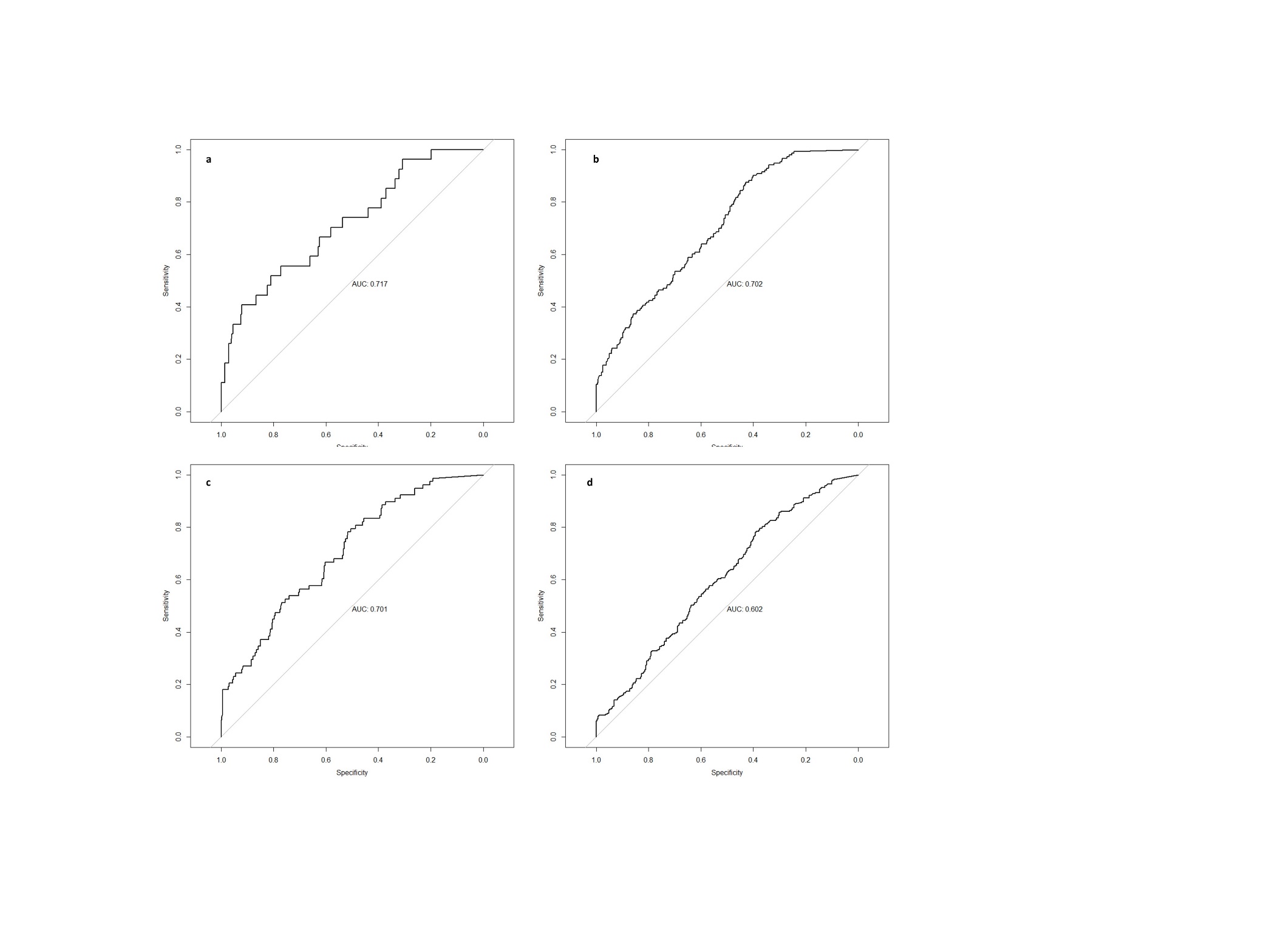


**S8 Fig.** The area under curve (AUC) statistic of the receiver operating characteristic curve for (a) Ag, (b) Wb123, (c) Bm14 and (d) Bm33 Abs
