# Supplementary material for: Spatial predictive risk mapping of lymphatic filariasis residual hotspots in American Samoa using demographic and environmental factors
Source: PLoS Negl Trop Dis. 2023 Jul 24;17(7):e0010840. doi: 10.1371/journal.pntd.0010840 (PMC10399813; doi:10.1371/journal.pntd.0010840)
Supplement: S1 Text — All of the parameters in this code were incorporated in the models for Wb123, Bm14 and Bm33 Ab. (DOCX) [file pntd.0010840.s011.docx]

**S1 Text.** OpenBuGS code of Bayesian geostatistical models for the probability of being positive for antigen in American Samoa, 2016. All of the parameters in this code were incorporated in the models for Wb123, Bm14 and Bm33 Ab.

model

{

for(i in 1:2671) {

Ag[i] ~ dbern(p[i])

logit(p[i]) <- V[Loc[i]] + beta[2]*age[i]+ beta[3]*female[i]+ beta[4]*outdoor[i] + beta[5]*tuna_cannery[i]+beta[6]*other[i]

}

for (j in 1:736){

V[j] <- W[j] + X[j] + Y[j]

W[j] <- beta[1]

X[j] <- beta[7]*Elev[j] + beta[8]*Rain[j] + beta[9]*Pop_den[j] + beta[10]*Dist_s[j] + beta[11]*Crop[j] + beta[12]*Forest[j] + beta[13]*Urban[j]

Y[j] <- theta[j]

mu[j] <- 0

}

theta[1:736] ~ spatial.exp(mu[], x[], y[], tau, phi, 1)

**# model above extrapolated to locations where outcome not observed:**

for(j in 1:2650) {

theta.pr[j] ~ spatial.unipred(mu.pr[j], x.pr[j], y.pr[j], theta[])

Ag.pr[j] ~ dbern(p.pr[j])

logit(p.pr[j]) <- beta[1] + theta.pr[j] + beta[7]*Elev.pr[j] + beta[8]*Rain.pr[j] + beta[9]*Pop_den.pr[j] + beta[10]*Dist_s.pr[j] + beta[11]*Crop.pr[j] + beta[12]*Forest.pr[j] + beta[13]*Urban.pr[j]

mu.pr[j] <- 0

}

for(i in 1:13){

beta[i] ~ dnorm(0.0,1.0E-3)

OR[i] <-exp(beta[i])

}

tau ~ dgamma(0.001,0.001)

sigma<- 1/tau

phi ~ dunif(0.03, 100)

}

list(

beta = c(

0.0,0.0,0.0,

0.0,0.0,0.0,0.0,0.0,

0.0,0.0,0.0,0.0,0.0), tau=1)
