# Supplementary material for: Spatial predictive risk mapping of lymphatic filariasis residual hotspots in American Samoa using demographic and environmental factors
Source: PLoS Negl Trop Dis. 2023 Jul 24;17(7):e0010840. doi: 10.1371/journal.pntd.0010840 (PMC10399813; doi:10.1371/journal.pntd.0010840)
Supplement: Attachment — Submitted filename: Response to reviewers.docx [file pntd.0010840.s012.docx]

**Response to reviewers**

Key Review Criteria Required for Acceptance?

**A. Methods**

*-Are the objectives of the study clearly articulated with a clear testable hypothesis stated?*

*-Is the study design appropriate to address the stated objectives?*

*-Is the population clearly described and appropriate for the hypothesis being tested?*

*-Is the sample size sufficient to ensure adequate power to address the hypothesis being tested?*

*-Were correct statistical analysis methods used to support conclusions?*

*-Are there concerns about ethical or regulatory requirements being met?*

- **Reviewer 1**

1. *L310: Separate models were fitted to different infection markers. Is it possible to just fit one model using the true prevalence rate as a latent variable and the prevalence rate of these four infection markers as different sets of observations of it. This is just a suggestion, no need to incorporate in the current manuscript.*

**Response:** One of the aims of this study was to build separate predictive models for different LF infection markers to predict probability for each of them and assess their geographical distribution. The main reason for this approach is that, based on existing data, each of the infection markers may be indicators of different stages of infection (1-4). There is no overall ‘true prevalence’ rate as such. Therefore, in this study we aimed to address the current need to investigate if Abs could potentially be used instead of, or in addition to Ag, in LF surveillance.

We acknowledge that further research is needed to improve the interpretation of these results and better understand role of the different infection markers in surveillance.

More information clarifying the meaning of Ag and the filarial antibodies has been added to the introduction (Lines 140 to 145).

“Currently, TAS relies on the monitoring of antigenemia in children aged 6 to 7 years (18); however, since Ag specific antifilarial antibodies such as Wb123, Bm14 and Bm13, generally develop before patent infection and may be indicators of different infection stages (LF exposure or infection patterns) their use in post-MDA surveillance could help to provide an early measure of filarial exposure and ongoing transmission (19)”.

1. Cadavid Restrepo AM, Gass K, Won KY, Sheel M, Robinson K, Graves PM, Fuimaono S, Lau CL. Potential use of antibodies to provide an earlier indication of lymphatic filariasis resurgence in post–mass drug administration surveillance in American Samoa. International Journal of Infectious Diseases. 2022 Apr 1;117:378-86.

2. Lau CL, Sheel M, Gass K, Fuimaono S, David MC, Won KY, Sheridan S, Graves PM. Potential strategies for strengthening surveillance of lymphatic filariasis in American Samoa after mass drug administration: Reducing ‘number needed to test’by targeting older age groups, hotspots, and household members of infected persons. PLoS neglected tropical diseases. 2020 Dec 28;14(12):e0008916.

3. Won KY, Sambou S, Barry A, Robinson K, Jaye M, Sanneh B, Sanyang A, Gass K, Lammie PJ, Rebollo M. Use of antibody tools to provide serologic evidence of elimination of lymphatic filariasis in the Gambia. The American journal of tropical medicine and hygiene. 2018 Jan;98(1):15.

4. Weil GJ, Kastens W, Susapu M, Laney SJ, Williams SA, King CL, Kazura JW, Bockarie MJ. The impact of repeated rounds of mass drug administration with diethylcarbamazine plus albendazole on bancroftian filariasis in Papua New Guinea. PLoS Neglected Tropical Diseases. 2008 Dec 9;2(12):e344.

2. *L313-6: These sentences are confusing. Am I understanding correctly that L312-4 describes the non-spatial model and L314-6 describe the spatial model, and the only difference is if the spatial random effect sj is included? I think it’s not fair to refer the model as “geostatistical model” if the spatial term is not included. The sentences could be modified to something like “We first developed a non-spatial model with the sociodemographic….. as the fixed effects, then extended this model to be a spatial model by including a spatial random effect term sj (Equation 1).”*

**Response:** The change has been made (Lines 331 to 332)

“First, non-spatial models were developed with the sociodemographic and environmental covariates as fixed effects but without considering the spatial dependence of the data. Then, Bayesian geostatistical models for each infection marker were fitted”

3. *L316-18: The statement about MCMC does not feel correct. Other methods, such as Restricted maximum likelihood can also accomplish these.*

**Response:** We did not mean to imply that Markov chain Monte Carlo (MCMC) are the only methods that allow incorporation of spatial dependence in the data, and that provides full representation of uncertainty in model outputs. In order to clarify the statement, the following change has been made (Lines 334 to 335):

“Then, Bayesian geostatistical models for each infection marker were fitted using a Markov chain Monte Carlo (MCMC) simulation approach with Gibbs sampling.”

4. *L331: (1) Why assuming a linear relationship between age and the prevalence rate?*

**Response:** We developed binomial models using positive/negative status of each infection marker as the outcome variable. We did not use prevalence rates. Quadratic terms for predictor variables were explored during the variable selection process. However, these terms did not improve the fit of the model. This has been specified in the manuscript with the following sentence in lines 324 to 325 and Lines 427 to 428.

“Nonlinear associations between predictors and the outcome variables were modelled using quadratic terms.”

“The quadratic terms did not improve model fit and were not included in the final models”.

*5. Since you already have household information, why not including it in the model? The* *infection status of people living in the same household are unlikely to be independent.*

**Response:** the geostatistical analyses were conducted at the household level. As the reviewer has indicated, we also believe that the infection status of people living in the same household and neighbouring houses are unlikely to be independent. Therefore, all models were developed considering the spatial dependence structure of the data and incorporated a geostatistical random effect (as a function of the separating distance between pairs of points). This information is specified in line 363.

No changes have been made.

*6. Several variables are included for occupation explicitly. What the reference level is? If Occupation is included in the model as a categorical variable, this should be done implicitly by the regression function when generating the design matrix. Moreover, the categories mentioned here are different from those presented in Table 3.*

**Response:** Multiple models were developed when exploring and analysing the data. In order to be consistent with previous work, in the final model, the occupation groups were categorised into those who worked i) predominantly indoors, ii) predominantly outdoors, iii) at the tuna cannery, and iv) others (including unemployed, unknown occupation, and those who have jobs that include both indoor and outdoor work). The mathematical notation has been updated (Lines 350 to 353):

$$Y_{ij} \sim Bern(p_{ij})$$

$$\mathrm{logit}\left( p_{ij} \right)= \alpha+{{\gamma\mathsf{x}\mathrm{age}}}_{i}+\delta\times\mathrm{female}_{i}+\varepsilon\times\mathrm{outdoor}_{i}+\eta\times{tuna cannery}_{i}+\theta\times\mathrm{others}_{i}+\sum_{z=1}^{z} \beta_{z} {\mathsf{x} \lambda}_{zj}+ s_{j}$$

The reference category for Work location was the category: “Indoor” as it is indicated in tables 3 and 4.

7. *L351: The convergence plots could be added to the appendix.*

**Response:** The plots have now been added to the supplementary material (Figs. S4 to S7)

8. *L364: What’s the purpose for using Bayesian kriging? Just for visualizing the results at a finer scale? If so, I think it is unnecessary given the low printing resolution anyway.*

**Response:** kriging was used to produce spatially smoothed maps of the predicted probability of LF infection markers throughout the study area.

High resolution maps (>300 dpi) have already been submitted. No changes have been made.

- **Reviewer 2**

1. *The study objectives are clearly stated.*

**Response:** No changes have been made.

2. *The population and sample size need to be clarified in the method section.*

**Response:** this information has been added to the updated manuscript in lines 220 to 222).

“A sample size of 4,620 was estimated to be required to detect Ag prevalence of 1% for persons aged ≥8 years. The total population of ~55,000 persons was assumed for American Samoa based on census data.

3. *Please clarify the description of Fig 2, what is K x 0.02 (line 276).*

**Response:** this means “Kelvin with scale factor set to 0.02”. However, the figure has been modified to exclude the temperature layer since these data were not included in the non-spatial or spatial models.

4. *It is not clear to me why the author excluded the temperature data from analyses. The author used rasters of rainfall and temperature with the same resolution at 1 km, but it turns out that temperature data wet not available for large areas (lines 392 to 393).*

**Response:** Temperature and rainfall layers were available at the same spatial resolution, 1km, but the coverage of the American Samoa territory was different. The data sources for these layers were different, as was the temporal resolution. Temperature layers were available weekly and needed more processing to calculate monthly and annual averages. Unfortunately, there were large areas with missing data in the temperature layers (figures below), and also lack of consistency in their coverage of the American Samoa territory. Therefore, we were not confident to include the temperature average estimates in the models. Below is an example of three temperature layers from different months. We included this issue as a limitation of the study in the Discussion.

No changes have been made.

- **Reviewer 3:**

1. *“Predictive” seems to be a misnomer in the title. The bulk of the modeling is association-based, with the spatial prediction occurring with only environmental data/population density at unsampled points. Crucially, no model validation was conducted. Why? The author could use a subset of sampled households/individuals and test on the remainder. Instead, prediction is made on unsampled continuous space. How much of that is inhabited? The authors never provide justification for this approach, as the Bayesian kriging is mostly reproducing the patterns from survey results.*

We disagree with the reviewer and believe that in this study we have implemented spatial methods to predict predicted probability in unsampled locations. We have added the word “Spatial” to the title to make this point clear.

Also, the models were developed again as suggested by the reviewer to be able to validate the results. This process is specified in the updated manuscript (Lines 390 to 396).

“To determine the predictive performance of the models of predicted probability of each infection marker, a validation dataset was created by random selection of 25% of the data. The ability of the final models to predict the probability of Ag, Wb123, Bm14 and Bm33 Abs was assessed by comparing the predicted probability of each infection marker to the observed results of the infection markers (0=negative, 1=positive). The area under curve (AUC) statistic of the receiver operating characteristic curve was used for the comparison. An AUC estimate of 0.7 was defined to indicate acceptable predictive performance”.

**B. Results**

*-Does the analysis presented match the analysis plan?*

*-Are the results clearly and completely presented?*

*-Are the figures (Tables, Images) of sufficient quality for clarity?*

- **Reviewer 1:**

1. *L374-7: Why the prevalence rates vary remarkably across infection markers?*

**Response:** existing data indicates that the infection markers may reflect different stages of LF infection (1-4). Therefore, the positivity and prevalence of Ag and Abs are expected to vary over time and across endemic areas (1-4). However, as it has been indicated in the manuscript, the kinetics of Ag and serological responses to Bm14, Bm33, and Wb123 are still poorly understood. Therefore, we believe that our results make a significant contribution to the literature on the spatial patterns of these infection markers and the potential value and challenges with using them in post-validation surveillance in *W. bancrofti*–endemic areas.

1. Cadavid Restrepo AM, Gass K, Won KY, Sheel M, Robinson K, Graves PM, Fuimaono S, Lau CL. Potential use of antibodies to provide an earlier indication of lymphatic filariasis resurgence in post–mass drug administration surveillance in American Samoa. International Journal of Infectious Diseases. 2022 Apr 1;117:378-86.

2. Lau CL, Sheel M, Gass K, Fuimaono S, David MC, Won KY, Sheridan S, Graves PM. Potential strategies for strengthening surveillance of lymphatic filariasis in American Samoa after mass drug administration: Reducing ‘number needed to test’by targeting older age groups, hotspots, and household members of infected persons. PLoS neglected tropical diseases. 2020 Dec 28;14(12):e0008916.

3. Won KY, Sambou S, Barry A, Robinson K, Jaye M, Sanneh B, Sanyang A, Gass K, Lammie PJ, Rebollo M. Use of antibody tools to provide serologic evidence of elimination of lymphatic filariasis in the Gambia. The American journal of tropical medicine and hygiene. 2018 Jan;98(1):15.

4. Weil GJ, Kastens W, Susapu M, Laney SJ, Williams SA, King CL, Kazura JW, Bockarie MJ. The impact of repeated rounds of mass drug administration with diethylcarbamazine plus albendazole on bancroftian filariasis in Papua New Guinea. PLoS Neglected Tropical Diseases. 2008 Dec 9;2(12):e344.

Because this information has already been provided in the manuscript (lines 550 to 557), no changes have been made.

*2. L381-4: Are the prevalence rates by infection markers highly correlated? What are the distributions of them? The fact that “villages with high prevalence of Bm33 Ab were more widespread” might due to that the prevalence rate of Bm33 Ab are higher than other places in general, and it looks like it’s high everywhere in Fig 3f when using the color scale that is appropriate for the other infection markers.*

**Response:** The prevalence rates of the infection markers may be correlated. However, these prevalences were not incorporated in our models. The models’ outcomes were positive and negative responses to the infection markers. Current evidence suggests that Wb123 Ab may be the first detectable seromarker that induces an Ab response even without high levels of antigenemia (1). On the other hand, Bm33 Ab persists long-term, which may explain the high prevalence of this infection maker is widespread across villages in American Samoa and other endemic areas.

1. Hamlin KL, Moss DM, Priest JW, Roberts J, Kubofcik J, Gass K, Streit TG, Nutman TB, Eberhard ML, Lammie PJ. Longitudinal monitoring of the development of antifilarial antibodies and acquisition of Wuchereria bancrofti in a highly endemic area of Haiti. PLoS neglected tropical diseases. 2012 Dec 6;6(12):e1941.

No changes have been made.

*3. L394: A corrplot could be included to represent the correlation coefficients between variables.* [*https://cran.r-project.org/web/packages/corrplot/vignettes/corrplot-intro.html*](https://cran.r-project.org/web/packages/corrplot/vignettes/corrplot-intro.html)

**Response:** following the suggestion of the reviewer, a corrplot has been created and added to the supplementary material (S3 Fig.)

*4. Table 2: I think this table could be moved to the appendix since it does not provide any necessary information for understanding the model or interpretating the model results*

**Response:** we thank the reviewer for the suggestion; however, we believe that the table provides descriptive statistics that help contextualised the outputs of the spatial models.

No changes have been made.

*5. Table 3: What does the blue background refers too? Looks like it represents cells with a significant relationship. Then why not use different colours for positive and negative relationships.*

**Response:** The table footnotes indicate that the statistically significant odds ratios are highlighted in blue. Based on the reviewer’s suggestion we have coloured the cells blue and grey to indicate statistically significant positive and negative associations, respectively.

6. *Table 3&4: If spatial model always performs better, I think it is reasonable to just include the results for spatial model in the main text and put the results for the non-spatial model in the appendix.*

**Response:** Table 3 has been moved to the supplementary material following the suggestion of the reviewer (S2 Table).

7*. L419-22: I feel interpreting the spatial scale parameter as the size of cluster is unreasonable. It stands for the cluster size of uncontrolled confounders.*

**Response:** We did not calculate the size of the cluster based on the spatial scale. The estimation of the cluster size was based on the values of the decay parameter for spatial correlation (*Φ*). This parameter is one of the main outputs of the spatial model*.* The values are usually given in decimal degrees by the model; therefore, it is necessary to convert the units to km to make the parameter useful in practical terms. This information has already been provided in the manuscript in lines 454 to 456.

No changes have been made.

**Reviewer 2:**

1. *Fig 1. Please check the label of the elevation classification in the legend. The author used classes 0 and 0-100.*

**Response:** The change has been made.

2. *Fig 3 a) needs to be expanded to show the locations of sample villages. The author mentions many times the high prevalence in the northwest, particularly Fagali'i and Fagamalo. It would be clearer for the readers if you move Fig 3a) to the study area section and make it bigger.*

**Response:** The change suggested by the reviewer has been made and the maps of the geographical distribution of villages and survey locations were moved to Figure 1.

3. *Please check the numbers used in the paragraph (Pages 21-23) and Table 3.*

**Response:** The numbers in the paragraphs indicated and in table 3 have been checked.

No changes have been made.

- **Reviewer 3:**

1. *Additionally, and crucially, on the prediction part, the values found and displayed in Figure 3 (survey data) and Figure 4 (prediction) are two orders of magnitude apart. Why? This seems to be more than just a rescaling error, as the text includes % that confirm this difference. It is hard to square this difference.*

The models have been run again for validation purposes and the values in figure 4 are presented as predicted probability in the updated manuscript.

2. *Figures 2-4 as provided are too low resolution*.

**Response:** high resolution images (300 dpi) were submitted to the journal.

**C. Conclusions**

*-Are the conclusions supported by the data presented?*

*-Are the limitations of analysis clearly described?*

*-Do the authors discuss how these data can be helpful to advance our understanding of the topic under study?*

*-Is public health relevance addressed?*

- **Reviewer 1:**

No Response

- **Reviewer 2:**

*Page 32, please clarify the association between the prevalence and tree coverage. It seems like the results show that 95% CI of all ORs include 1.00 (Table 3 and 4).*

**Response:** The 95% confidence intervals were presented as 1.00 to be consistent with the number of decimal points in the tables. In the updated manuscript, these values have been changed for “95% CI=1.001 to 1.01” in Table 3 and S2 Table (supplementary material).

- **Reviewer 3:**

1. *There are some major gaps in interpretation that are never addressed. The meaning of Ag vs. Wb123Ab vs. Bm14Ab vs. Bm33Ab are never satisfactorily addressed and should be brought up in the Introduction since Results interpretation depends on knowing their differences. I understand there is literature elsewhere on this, but a paper should stand-alone to a certain degree, and this is never addressed herein.*

**Response:** The meaning and kinetics of the different LF infection markers is still not very well understood. However, more information has been added to the manuscript to clarify this point (Lines 140 to 145).

“Currently, TAS relies on the monitoring of antigenemia in children aged 6 to 7 years (18); however, since Ag specific antifilarial antibodies such as Wb123, Bm14 and Bm13, generally develop before patent infection and may be indicators of different stages of infection, and their use in post-MDA surveillance could help to provide an early measure of filarial exposure and ongoing transmission (19)”.

2. *The role of the mosquito is also not well described or addressed in this paper, as they are strongly influenced by the environmental layers and other covariates used in the models. Why not? If there is no data on mosquitoes, then say so but explain what it would contribute; if there is, it should be incorporated into the modeling.*

**Response:** we have followed the suggestion of the reviewer and have included this information on the limitations section since there was no mosquito data at the household level available for this analysis (Lines 615 to 623).

The geographical distribution of LF may be influenced by the effect of the environment on vector mosquito populations and dynamics. A previous study conducted in American Samoa at the village level found statistically significant associations between PCR-positive *Ae. polynesiensis*, the primary LF parasite vector in the territory, and human seroprevalence of Ag and Wb123 Ab (12). Mosquito data at the household or village level were not available. Therefore, further surveys specifically designed to collect higher resolution data on mosquito abundance and distribution are needed to assess their associations with environmental and sociodemographic covariates, and influence on seroprevalence of LF in humans”.

3. *On both of these points, contradictory discussion is offered. The authors comment that there is “widespread distribution of positive Bm33 Ab responses”, but then can go on to say that :Bm33Ab can be detected more than a year before other Ab responses and can decrease after MDA”. Which one is more influential on results interpretation? They seem to point in opposite directions. Re:mosquitoes, there is brief suggestion of the importance of day-biting and night-biting mosquitoes but it’s not clear which is more important in American Samoa (or if they matter in different areas). There is also a line about risk to tuna cannery workers based on time of day when they work, but that is never explained (is it during the day? at night more?).*

**Response:**

**-** We agree with the reviewer and think that the information seems contradictory. The statement has been removed and information has been added to better clarify this point (Lines 556 to 557).

“It has been observed that the clearance of the Abs is highly variable and can persist for years after treatment in some individuals (53).

- Also, we have clarified in the updated manuscript that *Ae. polynesiensis* is the main vector in American Samoa (Lines 618 to 619). This vector is a day-biting mosquito and this information was already in the manuscript (Line 530).

“A previous study conducted in American Samoa at the village level found statistically significant associations between PCR-positive *Ae. polynesiensis*, the primary LF parasite vector in the territory, and human seroprevalence of Ag and Wb123 Ab (12)”.

- A statement has been added to the manuscript to explain the potential high risk of LF for tuna cannery workers (Lines 573 to 575).

“Tuna cannery workers are typically of low socioeconomic status and their poor living conditions may increase their risk of exposure to the infection”.

4. *Other comments: why was rainfall non-association finding unexpected? Never explained.*

**Response:** Additional information has been added to the manuscript to explain why this finding is unexpected (Lines 586 to 595).

“Rainfall has been shown to be associated with high prevalence of LF in several endemic countries where the infection is transmitted by different vectors (22, 25, 33, 58, 59). Rainfall generates water pools that can serve as mosquito breeding sites and influence mosquito abundance and behaviour (60). In contrast, heavy rainfall may have a negative effect on LF prevalence by causing excessive damage to mosquito larval habitats (60). No associations between the prevalence of infection markers and rainfall were found in this study. This finding was unexpected and deserves further investigation. These findings raise the need for high-quality spatial environmental datasets that can be used in further studies to determine the association of LF and other potential environmental drivers”.

D. **Editorial and Data Presentation Modifications?**

*Use this section for editorial suggestions as well as relatively minor modifications of existing data that would enhance clarity. If the only modifications needed are minor and/or editorial, you may wish to recommend “Minor Revision” or “Accept”.*

- **Reviewer 1:**

No Response

- **Reviewer 2:**

Minor Revision

- **Reviewer 3:**

1. *123: Ag not defined yet but abbreviation used*

**Response:** the change has been made (Line 123).

“WHO-recommended school-based transmission assessment surveys (TAS) conducted in 2011 (TAS-1) and 2015 (TAS-2) with crude prevalence of antigen (Ag)-positive of 0.2% (95% confidence interval (CI) 0.0 to 0.8%) and 0.1% (95% CI 0.0 to 0.7%), respectively.

2. 126: *What is the threshold?*

**Response:** The threshold is already indicated in the manuscript (Lines 127). No changes have been made.

“higher than the threshold and the recommended upper 95% confidence limit of 1%”.

3. *130: What to make of other studies showing ongoing transmission while TAS-1 and TAS-2 were passed? Please comment.*

**Response:** This highlights the need for more sensitive, standardized tools to help programs define MDA endpoints and to conduct post-MDA surveillance.

This information has been added to the updated manuscript in lines 137 to 138.

“As LF prevalence decreases, the ability of diagnostic methods that are sufficiently sensitive, particularly in the TAS, to detect areas with residual transmission is also limited”.

4. *153-155: Sentence fragment, rewrite*

**Response:** the sentence has been modified (Lines 159 to 161).

“Bayesian model-based geostatistics incorporating infection prevalence data with socio-demographic and environmental covariates has proven to be able to predict disease distribution in areas with scarce information (24-26)”.

5. *Fig 2: Fix population density units label, convert temperature map to something more human readable (Celsius perhaps).*

**Response:** The figure has been edited and the temperature layer was removed since it was a variable not included in the model and that was causing some confusion for other reviewers.

6. *301-3: Rephrase this sentence (verb agreement)*

**Response:** The change has been made (Lines 319 to 322).

“For the strongly correlated covariates (Spearman’s correlation coefficient ρ > 0.8), the ones with the highest value of Akaike Information Criterion (AIC) in the univariate regression models were excluded (Supplementary Fig. S3)”.

7. *331: Starkist? Should you just say (Tuna) Cannery?*

**Response:** This word and the formula notation have been changed (Line 352).

$$Y_{ij} \sim Bern(p_{ij})$$

$$\mathrm{logit}\left( p_{ij} \right)= \alpha+{{\gamma\mathsf{x}\mathrm{age}}}_{i}+\delta\times\mathrm{female}_{i}+\varepsilon\times\mathrm{outdoor}_{i}+\eta\times{tuna cannery}_{i}+\theta\times\mathrm{others}_{i}+\sum_{z=1}^{z} \beta_{z} {\mathsf{x} \lambda}_{zj}+ s_{j}$$

8. *Page 30, etc.: Lowercase for species names (polynesiensis, bancrofti)*

**Response:** The changes have been made (Lines 529 to 530).

E. **Summary and General Comments**

*Use this section to provide overall comments, discuss strengths/weaknesses of the study, novelty, significance, general execution and scholarship. You may also include additional comments for the author, including concerns about dual publication, research ethics, or publication ethics. If requesting major revision, please articulate the new experiments that are needed.*

- **Reviewer 1:**

*This manuscript developed a Bayesian geostatistical model to examine the environmental determinants of lymphatic filariasis and predict its prevalence on a fine scale for American Samoa. In general, I think the method is valid and the results are well-presented. Some clarifications could improve the readability further. For example, what do the four infection markers represent and what are the relationships between them? Do they refer to infections caused by different types of parasites and are there systematic relationships between them?*

**Response:** These points are all reasonable and more information about the meaning of the different infection markers have been added to the updated manuscript (Lines 139 to 143)

- **Reviewer 2:**

*The Bayesian model-based geographics approach combines with sociodemographic and environmental data were used to predict the spatial distribution of LF prevalence in American Samoa. The manuscript was well written and will make a good contribution to the knowledge of LF epidemiology in American Samoa.*

- **Reviewer 3:**

*The motivating question for this paper is interesting; how do we use modeling to support more specific targeting of post-MDA surveillance activities? I don’t think the methods are sufficient though and the claim of being “predictive” is a misnomer. Model validation is a must; the orders-of-magnitude gulf between surveys and predicted values is not reassuring to readers. I would encourage the authors to rethink their approach.*

**Response:** We believe that there is predictive work conducted in the study. Validation has been conducted as suggested by the reviewer and results presented in the updated manuscript. Also, it has been clarified that the results are predictive probability estimates.
