# Supplementary material for: Spatial predictive risk mapping of lymphatic filariasis residual hotspots in American Samoa using demographic and environmental factors
Source: PLoS Negl Trop Dis. 2023 Jul 24;17(7):e0010840. doi: 10.1371/journal.pntd.0010840 (PMC10399813; doi:10.1371/journal.pntd.0010840)
Supplement: Attachment — Submitted filename: Response to reviewers.docx [file pntd.0010840.s013.docx]

**Response to reviewers**

Key Review Criteria Required for Acceptance?

**A. Methods**

*-Are the objectives of the study clearly articulated with a clear testable hypothesis stated?*

*-Is the study design appropriate to address the stated objectives?*

*-Is the population clearly described and appropriate for the hypothesis being tested?*

*-Is the sample size sufficient to ensure adequate power to address the hypothesis being tested?*

*-Were correct statistical analysis methods used to support conclusions?*

*-Are there concerns about ethical or regulatory requirements being met?*

- **Reviewer 1**

No comment

- **Reviewer 2**

1. *Do the study area information original from the author, if not please add appropriate references.*

**Response:** The paragraph has been appropriately referenced (lines 185 to 199).

“American Samoa is a United States territory in the South-central Pacific located approximately between latitudes 11° North and 15° South and longitudes 168° East and 172° West (Fig 1). The total land area of the territory is 200 km2 and comprises five inhabited volcanic islands Tutuila, Aunu’u, Ofu, Olosega and Ta’ū, and two remote coral atolls (Swains Island and Rose Atoll). In 2010, the population of American Samoa was 55,519, the majority of whom (95%) lived in Tutuila, the largest island (198.9 km2), where the capital Pago Pago is located (27).

American Samoa lies in the tropical savanna climate zone characterized by alternate wet (October to May) and dry (June to September) seasons (28). Temperatures vary slightly between the hottest period (December to April), when the average is approximately 31°C, and the coolest period (June to August), when the average is 29 °C. The annual average rainfall ranges from 3000 to 6000 mm, with 70% occurring during the hot and wet season (28). The average elevation is 482 meters (m) with the highest point being Lata Mountain on the island of Ta’ū (970 m) (29).”

2. *Line 220: The author stated that a sample size was 4620, how the author calculated the sample size? Why the sample size and number of participants are difference?*

**Response:** The original target sample size for the community-based survey was calculated based on an expected antigen prevalence of 1%. However, after the first two weeks of recruitment, the observed antigen prevalence (~4%) was significantly higher than anticipated, and it was determined that a smaller target sample size of 2,981 would provide adequate statistical power. A detailed description of the sampling design can be found here (1). This reference is provided in the manuscript. Also, information about the target sample size has been clarified in the updated manuscript (lines 215 to 225).

1. Sheel M, Sheridan S, Gass K, Won K, Fuimaono S, Kirk M, et al. Identifying residual transmission of lymphatic filariasis after mass drug administration: Comparing school-based versus community-based surveillance-American Samoa, 2016. PLoS neglected tropical diseases. 2018;12(7):e0006583.

“Based on a total population of 55,000 (27), a target sample size of 4,620 was estimated to be required to detect Ag prevalence of 1% for persons aged ≥8 years. The target number of households that needed to be visited was calculated by dividing the target sample size of persons by the average household size and accounting for a 15% non-response/ absentee rate (the most recent census provided the total numbers of households in the selected villages and indicated that the average of persons per household was seven). A sampling fraction of 0.29 was calculated as the proportion of households that needed to be sampled on each PSU to achieve the target sample size. However, after the first two weeks of recruitment, the observed antigen prevalence was approximately 4%, which is significantly higher than anticipated, and it was determined that a smaller target sample size of 2,981 would be sufficient to obtain adequate statistical power”.

3. *Please describe how many household members aged>8 years in 32 PSUs and 754 households in total and how many of them were surveyed?*

**Response:** Based on the clarification above about the target sample size (2,981) and the actual number of participants was 2,671, it is estimated that 89.6% of the target sample size were surveyed.

As indicated in comment 2, a more detailed description of the sampling design is provided in the updated version of the manuscript (lines 215 to 223).

“Based on a total population of 55,000 (27), a target sample size of 4,620 was estimated to be required to detect Ag prevalence of 1% for persons aged ≥8 years. The target number of households that needed to be visited was calculated by dividing the target sample size of persons by the average household size and accounting for a 15% non-response/ absentee rate (the most recent census provided the total numbers of households in the selected villages and indicated that the average of persons per household was seven). A sampling fraction of 0.29 was calculated as the proportion of households that needed to be sampled on each PSU to achieve the target sample size. However, after the first two weeks of recruitment, the observed antigen prevalence was approximately 4%, which is significantly higher than anticipated, and it was determined that a smaller target sample size of 2,981 would be sufficient to obtain adequate statistical power.”

**Reviewer 3:**

1. *The manuscript is improved from the earlier draft in that it attempts to perform model validation. However, what is described is basic and not sufficient. Cross validation is needed rather than removing 25% of the data one time.*

**Response:**

We thank the reviewer for the comment. However, we believe that the validation set approach used to validate the geostatistical models in this study is a valid validation procedure routinely used in several similar studies that involve spatial analyses and Bayesian geostatistics (1-5). From a Bayesian point of view, cross-validation using different sets of data or repeated data splitting can be computationally demanding, as estimation of parameters usually requires Monte Carlo Markov Chain (MCMC) methods (6). The usual setup for model validation in geostatistics is to make prediction for one or a few selected validation sets. Of note, prediction error or uncertainty is also estimated by implementing these models and is inferred from the predicted values. This is the reason why we have also mapped (in figure 4) the posterior standard deviation of the predicted probability of each infection marker.

No changes have been made.

1. Owada K, Lau CL, Leonardo L, Clements AC, Yakob L, Nielsen M, et al. Spatial distribution and populations at risk of A. lumbricoides and T. trichiura co-infections and infection intensity classes: an ecological study. Parasites & vectors. 2018;11(1):1-13.(4)

2.	Tsheten T, Clements AC, Gray DJ, Wangdi K. Dengue risk assessment using multicriteria decision analysis: A case study of Bhutan. PLoS neglected tropical diseases. 2021;15(2):e0009021.

3.	Soares Magalhães RJ, Salamat MS, Leonardo L, Gray DJ, Carabin H, Halton K, et al. Mapping the risk of soil-transmitted helminthic infections in the Philippines. PLoS Neglected Tropical Diseases. 2015;9(9):e0003915.

4.	Soares Magalhães RJ, Biritwum N-K, Gyapong JO, Brooker S, Zhang Y, Blair L, et al. Mapping helminth co-infection and co-intensity: geostatistical prediction in Ghana. PLoS neglected tropical diseases. 2011;5(6):e1200.

5.	Owada K, Nielsen M, Lau CL, Yakob L, Clements AC, Leonardo L, et al. Functional illiteracy burden in soil-transmitted helminth (STH) endemic regions of the Philippines: An ecological study and geographical prediction for 2017. PLoS neglected tropical diseases. 2019;13(6):e0007494.

6. Marshall E, Spiegelhalter D. Approximate cross‐validatory predictive checks in disease mapping models. Statistics in medicine. 2003;22(10):1649-60.

7. Noor AM, Clements AC, Gething PW, Moloney G, Borle M, Shewchuk T, et al. Spatial prediction of Plasmodium falciparum prevalence in Somalia. Malaria journal. 2008;7:1-13.

2. *Why was using intervention terms in the model not investigated or discussed?*

**Response:** This paper was focused on predicting the distribution based on sociodemographic and environmental factors. Adding intervention terms to the models was out of the scope of this study’s aims. We believe that this is a valuable suggestion that can be considered for future work.

3. *I am not sure why the sample size information is included when it was not attained to reach the power pre-specified. Is this a problem for this paper rather than the prior studies cited? Nonetheless, I do not dispute its inclusion since another reviewer requested it, but I do not see its added value.*

**Response:**

This information has not been removed since Reviewer 2 has requested more details about the sampling design. Therefore, a more detailed description of how the target sample size was estimated is now provided in the updated version of the manuscript (lines 215 to 223).

“Based on a total population of 55,000 (27), a target sample size of 4,620 was estimated to be required to detect Ag prevalence of 1% for persons aged ≥8 years. The target number of households that needed to be visited was calculated by dividing the target sample size of persons by the average household size and accounting for a 15% non-response/ absentee rate (the most recent census provided the total numbers of households in the selected villages and indicated that the average of persons per household was seven). A sampling fraction of 0.29 was calculated as the proportion of households that needed to be sampled on each PSU to achieve the target sample size. However, after the first two weeks of recruitment, the observed antigen prevalence was approximately 4%, which is significantly higher than anticipated, and it was determined that a smaller target sample size of 2,981 would be sufficient to obtain adequate statistical power.”

**B. Results**

*-Does the analysis presented match the analysis plan?*

*-Are the results clearly and completely presented?*

*-Are the figures (Tables, Images) of sufficient quality for clarity?*

- **Reviewer 1:**

No comment

- **Reviewer 2:**

I have no further comment in the result section

- **Reviewer 3:**

1. *The AUC values are not compelling, and I would like to see it done with cross validation, including reporting of variance. Three markers are around 0.7 (the threshold chosen by the authors but not an impressive one), and another is close to 0.6. If the best models are still not performing well, that is okay to report and publish, but the text/results on AUC are key to the paper and haven't been appropriately treated in this revision.*

**Response:** As it has been indicated in Section A (Methods), in Bayesian geostatistics, cross-validation (K-fold cross-validation and repeated data splitting) can be computationally very demanding since a full MCMC analysis has to be repeated multiple times. The validation set approach is a standard method used when applying Bayesian geostatistics. In this study, we categorised the area under the curve of the receiver operating characteristic based on the discriminatory performance of the models relative to the observed status at validation locations. The AUC threshold selected followed what has been suggested by Brooker et al., 2002 (1):

- AUC values between 0.50 and 0.69 = poor discriminative capacity in distinguishing between different populations.
- AUC from 0.70–0.89 = reasonable capacity
- AUC values >0.90 = very good capacity

Based on the proposed thresholds, we concluded that most models have a reasonable predictive performance.

More detailed information has been added to the updated manuscript (lines 411 to 418 and lines 530 to 535):

“The models for Ag, Wb123 and Bm14 Abs were able to predict the probabilities of these outcomes reasonably well with AUC values of >0.71, 0.70 and 0.70, respectively. However, the AUC value for the model of Bm33 Ab performed poorly with an AUC value of 0.602 (Supplementary Fig.8). Of all the infection markers investigated, positive Bm33 Ab was the most widespread, and the poor performance of this model could indicate that sociodemographic and environmental factors may not be important determinants of its geographic distribution”.

1. Brooker S, Hay SI, Bundy DA. Tools from ecology: useful for evaluating infection risk models? Trends in parasitology. 2002;18(2):70-4.

2. *- S4-S7: For these plots, it is not clear by visual inspection that convergence is achieved. Are these the wrong 10,000 values shown? You are fitting a lot of parameters.*

**Response:** Because there is no global or standard diagnostics test to assess convergence of MCMC simulations, we assessed convergence by visual inspection of history plots of the chains for the regression coefficient variables (1). We disagree with the reviewer and we are confident that convergence has been achieved since most chains in the history plots appear to be overlapping one another and show appropriate mixing (1).

1. Spiegelhalter D, Thomas A, Best N, Lunn D. OpenBUGS user manual. Version. 2007;3(2):2007

*- How do we know to not be concerned about overfitting? Please check*

As the reviewer has indicated before, the AUC values show that most models performed reasonably well (and one performed poorly).

No changes have been made.

**C. Conclusions**

*-Are the conclusions supported by the data presented?*

*-Are the limitations of analysis clearly described?*

*-Do the authors discuss how these data can be helpful to advance our understanding of the topic under study?*

*-Is public health relevance addressed?*

- **Reviewer 1:**

No comment

- **Reviewer 2:**

No comment

- **Reviewer 3:**

1. *The model results (significant terms, DIC, AUC, etc.) vary quite a bit by antibody, but the authors do not spend significant time/text discussing this or thinking about the interpretation of results. A reviewer from the prior draft also brought this up, and the authors have not adequately addressed this point despite starting to sketch out some important points in their response without modifying the text. To get to public health relevance, the relationships need to be understood, and model results should at least accord with biological understanding, or commentary should be made about the mismatch.*

**Response:** As indicated in the previous review, the evidence is clear in that LF infection markers behave differently at different stages of LF infection. Therefore, it is expected that the detection of positive cases and prevalence of Ag and Abs vary over time and across endemic areas. However, how and when the production and clearance of Ag and Abs occurs and how these infection makers behave post-MDA is still not clear. As a result, the observed variation in AIC, DIC, AUC, etc. of the different models was expected.

Significant associations also varied slightly between infection markers. However, longitudinal studies are required, as we indicated in the manuscript, so that we can provide precise biological interpretations.

More information has been added to the updated manuscript in lines 596 to 608.

“In this study, the geographical distribution of the predicted prevalence of Wb123 and Bm14 Abs were more clustered compared to the widespread distribution of positive Bm33 Ab responses. It has been observed that Abs can be detected earlier and are higher in prevalence when compared to Ag (57, 58). Also, Ab clearance is highly variable and can persist for years after treatment in some individuals (57). This finding suggests that Bm33 Ab may not be the best indicator to identify areas of ongoing *W. bancrofti* transmission but may be used to provide information about levels of historical exposure and infection. It has been observed that the clearance of the Abs is highly variable and can persist for years after treatment in some individuals (57). This finding also concurred with a recent study indicating that the combined use of Ag and Bm14 Ab provides a more sensitive marker of current or previous infection than each indicator alone (59). Additional longitudinal studies are required to help monitor how the stage of the infection and magnitude of the immunological responses determine the spatial patterns of antifilarial Abs. Such information will have implications for the selection of the most suitable LF diagnostic tools in low prevalence and post-MDA settings”.

D. **Editorial and Data Presentation Modifications?**

*Use this section for editorial suggestions as well as relatively minor modifications of existing data that would enhance clarity. If the only modifications needed are minor and/or editorial, you may wish to recommend “Minor Revision” or “Accept”.*

- **Reviewer 1:**

No comment

- **Reviewer 2:**

No comment

- **Reviewer 3:**

1. *Conversion of cluster size inexact (line 483) because American Samoa is not at equator. Please use a better method.*

**Response:** The authors agree with the reviewer in that American Samoa is 14 degrees south of the equator. However, this metric is an approximate conversion. It may not correct precisely for the Earth's polar flattening but the error is probably negligible for the purpose of calculating cluster size. Therefore, no changes have been made.

2. *S4: Why only 6,000 values instead of 10,000? Please correct.*

**Response:** History plots showing 10,000 iterations have now been provided all models.

E. **Summary and General Comments**

*Use this section to provide overall comments, discuss strengths/weaknesses of the study, novelty, significance, general execution and scholarship. You may also include additional comments for the author, including concerns about dual publication, research ethics, or publication ethics. If requesting major revision, please articulate the new experiments that are needed.*

- **Reviewer 1:**

No comment

- **Reviewer 2:**

No comment

- **Reviewer 3:**

*The manuscript is improved from the prior draft but still needs major improvements related to cross validation, checking convergence and overfitting, and interpretation of model results for public health relevance meaningfully for readers.*

*Data should be made publicly available so results can be replicated. Ideally, code would also be shared so reviewers/future readers can have confidence in the results and conclusions presented herein*

**Response:** All the responses to the reviewer’s comments in relation to validation, convergence, overfitting and interpretation have been addressed in their respective sections.

As indicated during the submission process the data cannot be made available since that would represent a breach of confidentiality. However, the code for the geostatistical model has now been added to the supplementary material.
